# Exploring the associations between auditory hallucinations and psychopathological experiences in 10,933 patient narratives: moving beyond diagnostic categories and surveys

**DOI:** 10.1186/s12888-023-04780-2

**Published:** 2023-05-02

**Authors:** Chandril Chandan Ghosh, Duncan McVicar, Gavin Davidson, Ciaran Shannon, Cherie Armour

**Affiliations:** 1 School of Psychology, Queen’s University Belfast, Belfast, UK; 2 Queen’s Management School, Queen’s University Belfast, Belfast, UK; 3 School of Social Sciences, Education and Social Work, Queen’s University Belfast, Belfast, UK; 4 IMPACT Research Centre, Northern Health and Social Care Trust, Antrim, UK

**Keywords:** Auditory hallucination, Psychosis, Symptomics, Narrative, Correlation

## Abstract

**Background:**

Previous research suggests that auditory hallucinations are prevalent within both the clinical and general populations. Yet, we know little about how these phenomena are associated with other psychopathology symptoms and experiences. The current study aids investigations towards preventing, predicting and more effectively responding to such distressing occurrences. There have been substantial efforts in the literature to propose models of auditory hallucination and attempts to verify them. However, many of these studies used survey methods that restrict the person’s responses to a set of pre-defined criteria or experiences and do not allow exploration of potential important other symptoms beyond them. This is the first study to explore the correlates of auditory hallucination using a qualitative dataset consisting of unrestricted responses of patients about their lived experiences with mental illness.

**Method:**

The study used a dataset consisting of 10,933 narratives from patients diagnosed with mental illnesses. For analysis, the study used correlation on the text-based data. This approach is an alternative to the knowledge-based approach where experts manually read the narratives and infer the rules and relationships from the dataset.

**Result:**

This study found at least 8 correlates of auditory hallucination (small correlation coefficients), with the unusual ones being “pain.” The study also found that auditory hallucinations were independent of obsessive thoughts and compulsive behaviours, and dissociation, in contrast with the literature.

**Conclusion:**

This study presents an innovative approach to explore the possible associations between symptoms without the restrictions of (or outside the confines of) traditional diagnostic categories. The study exemplified this by finding the correlates of auditory hallucination. However, any other symptom or experience of interest can be studied similarly. Potential future directions of these findings are discussed in the context of mental healthcare screening and treatment.

**Supplementary Information:**

The online version contains supplementary material available at 10.1186/s12888-023-04780-2.

## Background

Auditory hallucinations are a cardinal feature of schizophrenia spectrum disorders. They have often been linked to suicide attempts [1] and homicide [2]. However, they are not disease-specific [3]. Furthermore, they have been reported in 10–15% of the non-clinical population in individuals with no clinical diagnosis. Hence, such experiences are regarded as relatively common.

On the other hand, auditory hallucinations can be indicative of an organic cause such as a brain tumour [4], and non-organic psychiatric illness such as psychotic, affective, and personality disorders [5]. However, despite such prevalence and associations with serious illnesses, there is little research focusing on what symptoms tend to co-occur with auditory hallucination, which can further inform understanding of the onset and maintenance of these potentially distressing experiences. Additionally, such knowledge could help identify specific patterns of symptoms that may not conform to traditional diagnostic categories and enable more individualised and effective responses.

### Theoretical frameworks for auditory hallucination

Auditory hallucinations are often associated with other psychopathological experiences. In terms of lifetime prevalence, a broad pattern has previously been identified, with auditory hallucinations being 2-3-fold more common than visual hallucinations, which are around twice as common as tactile hallucinations, which in turn were around twice as common as olfactory hallucinations [6]. In addition, some of the major theoretical frameworks that describe the psychological mechanisms underlying hallucinations suggest that the voice’s appraisal (meaning, identity, and supposed malevolent intention) was closely linked to distress [7]. For example, beliefs of “malevolent” intent and the power of the voices are considered to trigger anxiety and depression [8].

A model proposed that abnormal neural activation patterns lead to experiences of auditory hallucinations. Such activation patterns produce auditory signals linked to errors in signal detection, deficits in executive and inhibition, memories and expectations and other factors which influence how such events or experiences are interpreted [9].

Emotional factors play a particularly prominent role at all levels of this hierarchy. For example, anxiety could result from beliefs about the voices and, at the same time, facilitate the recurrence of auditory hallucinations and thus be a perpetuating factor [7]. Therefore, auditory hallucinations have been argued to trigger a new set of symptoms (e.g., those related to anxiety syndrome). Alternatively, other psychopathological symptoms such as those related to anxiety can also induce verbal hallucinations [10]. Therefore, auditory hallucination could be related to other psychopathological symptoms and experiences in both directions. The literature needs to discover the complete set of symptoms and experiences that are associated with auditory hallucination for a more comprehensive understanding of the phenomenon of hearing voices.

### The traditional approach to studying auditory hallucination and psychopathology in general

Conventionally, auditory hallucinations, like most other phenomena in mental ill-health experiences, had been frequently studied in the context of the DSM and ICD based categories of mental disorders such as schizophrenia and related psychoses, or on their early signs and symptoms in high-risk “prodromal” groups e.g., [11, 12].

The limitations of this category-based approach to diagnosis and treatment are increasingly being recognised e.g., [13]. There tend to be several main areas of concern about these traditional approaches. First, the diagnostic categories suffer from high diagnostic heterogeneity, indicating that two patients diagnosed with the same disorder have different symptomatic experiences [14]. Second, this problem does not disappear when Artificial Intelligence does the clustering instead of humans [15]. Third, both of these studies utilised the same dataset used in the current study consisting of patients’ first-hand narratives about their lived experiences. Therefore, the concerns are related to the description of mental illnesses.

### A new approach – shifting the object of inquiry to individual symptoms

It has been proposed that psychopathology be studied using individual symptoms as the object of analysis [16] instead of using diagnostic categories. This line of emerging literature mostly attempts to describe psychopathological experiences.

Current studies using symptoms as the object of analysis have progressed using new lines of analytics such as the network approach (N.A.) e.g., [17–20]. One of the advantages of N.A. is that you can mathematically analyse and visualise the interrelationship of symptoms. From a topological point of view, the network structure is composed of nodes representing the variables studied and the edges or lines connecting the nodes and representing their relationship. In addition, graph theory has been used to represent different spatial and functional characteristics that reveal information about the types of relationships between the network nodes.

### Problem with the new approach – the source of data

The findings of data analysis are dependent on the quality of the data that is fed in. Many of these symptomic studies have used questionnaires to collect data similar to the previous studies using DSM categories. However, there are several concerns with using such tools for data collection. For example, such questionnaires intend to measure questionable constructs, such as depression which consists of different items/symptoms for different experts. So, there is no consensus on which symptoms to consider; for example, there is little overlap between some aspects of the most used depression questionnaires [21].

Additionally, there is an underlying assumption that all symptoms (represented by items) contribute towards estimating the construct with equal weight, which is often not the case. Furthermore, using questionnaires also means that the respondent is often restricted to respond to only a few symptoms based on the researchers’ choice of standardising measure– and so maybe leaving out other symptoms even if they are more frequent and important to the respondent (other symptoms which may or may not be covered in a different psychometric measure of the particular construct had been chosen). Together, these issues raise an important question: which symptoms to consider (and which not to) while exploring a particular phenomenon, syndrome, or symptomatic experience? Ideally, one would want to query respondents regarding all the unique and potential symptoms from across all the relevant questionnaires, but that is not feasible/practical. So, while such a comprehensive approach would address one concern (i.e., the appropriate object of inquiry), other concerns remain unresolved (i.e., how to collect all the relevant data).

### Potential solution

We propose that the solution lies in investigating symptoms of mental illnesses using rich, unrestrictive textual narratives of lived experiences from patients diagnosed with mental illnesses – as an alternative to survey data. In recent studies [14, 15], we attempted to explore the network structure of psychopathological symptoms and experiences using rich, unrestrictive textual narratives of lived experiences from patients diagnosed with mental illnesses. Both of these studies utilised the same data we used in the current study. It was found that ‘auditory hallucinations’ was one of the most frequently co-occurring experiences among all other symptoms of psychopathology, indicating a transdiagnostic nature. However, given the textual nature of data, the relationships were based on co-occurrences, and it did not communicate the strength of such relationships.

The current study investigates the strength of such symptomatic associations of auditory hallucination with other psychopathological experiences. In addition to contributing to the theoretical advancement of the psychosis literature, the process demonstrates how to use text-based data and find co-occurrences as an alternative to using survey-based data and find correlation coefficients.

A previous study used Network Analysis to identify the co-occurring symptoms of psychopathology [15] from the patients’ narrative data. However, it did not convey information about “how strong?” or “interesting” the relationships were. This is the first study that demonstrates how to find the numeric strength of associations between co-occurring symptoms from a purely text-based database narrating patients’ experience. We used correlation analysis to find associations between symptoms from web-scraped narrative data in the mental health literature to achieve this. Therefore, this study demonstrates how we can quantify rich qualitative data sources and employ standard statistical analysis.

Borrowing the idea from machine learning literature, we propose three main stages of developing research in this emerging area: Describe, Predict and Prescribe. Much of the emerging symptomic literature on mental ill-health, including the current study, is expected to contribute to the exploration/description (of psychopathological experiences) stage. Once this stage of research is sufficiently well developed and we know which symptoms or experiences are inter-related or co-varies, based on such findings, future studies could use such relationships and attempt to predict one symptom from the other(s) and contribute towards better prognosis, screening, and preventive interventions. Once we can predict a condition or experience from others, it opens up possibilities to prescribe interventions. Therefore, in the third and final stage, the research could be developed to inform prescribing clinicians and other stakeholders on which treatment, initiatives, or interventions to offer, to whom and in what circumstances using the predictive relations. As mentioned above, this study is expected to inform the first stage of mental healthcare research, describing the relationships between conditions or experiences.

### Research questions

In this study, we attempted to explore the association of auditory hallucination with other narrative descriptions of psychopathological symptoms. This is expected to contribute towards developing our understanding of auditory hallucinations. The merit of the current study lies in the fact that it uses a source of data that does not restrict patients’ responses to a pre-fixed set of questions, symptoms or conditions. In contrast, the typical method of data collection in mental health literature involves setting a list of variables, using scales to prompt respondents to answer those variables, and then conducting correlation analysis. This survey-based approach poses a risk of confirmation bias since the researcher already decides which symptoms to investigate before collecting data. Furthermore, if a questionnaire only covers nine depression symptoms (such as the PHQ-9) but a patient experiences fifteen different symptoms, there is no opportunity for the patient to report the additional six symptoms, leading to potential data loss.

On the other hand, in data sources such as narratives and interview transcripts, the researcher either asks open-ended questions or does not ask anything at all. Either way, the participant gets the opportunity to express all the symptoms they want, and the researcher then tries to find associations between all the reported symptoms after the data collection is over. In simpler words, in survey data, the symptoms are given by the researcher. In narrative or interview data, the patient or participant gives the symptoms, implying an association between the participants’ reported symptoms.

Furthermore, the surveys using Likert scales are susceptible to response biases such as social desirability and acquiescent responding - comprising with the compromise the fairness and the validity of the assessments [22].

On the other hand, the data source we used in the current study offered the usage of fictitious identity. Since no one is approaching the patients (such as the researcher or someone in authority), we argue that they are more likely to be open and honest with their responses because they are the ones who are voluntarily logging into the forum and writing about their experience. Finally, the size of the data and the economic factors associated with it is another merit of the data and method we used. About 10,000 + data were collected within one minute. Thus, the reason why the current study needs to exist is to demonstrate an alternative approach to collect data and use correlation on it without running the list of missing out on variables that are actually important to the patient; reducing the likelihood of potential biases among the respondents, and collect a large dataset within a few short periods of time free of cost using a personal computer.

Additionally, in the current study, we also aim to find the variables that were independent of auditory hallucinations (correlation coefficient = 0). Still, in contrast, the literature suggested an association. In line with the above discussion, such differences might be important to note. At the least, it raises a question about the proposed relationship and urges further investigations towards confirmation.

## Method

### Data

The current study utilised a dataset consisting of 10,933 narratives from patients diagnosed with mental illnesses and their lived experiences. It is secondary data for the current study because we collected it in a previous study [14]. In this prior study we proposed an alternative approach to measuring the reliability of the diagnostic system in mental health. The researchers used Jaccard’s similarity index analysis on 228 narratives from patients with mental disorders to demonstrate the high heterogeneity and limited reliability of the existing categorical taxonomic system (e.g. DSM). The results showed that narratives are a statistically viable data resource and can distinguish between patients with different diagnostic labels, but the similarity coefficients between the majority of narrative pairs were low. This study highlighted the need for a more comprehensive and accurate diagnostic system in mental health and has potential applications in healthcare management and mental health research.

The data was sourced from an online platform called https://www.livejournal.com/. The data is devoid of any numeric responses, such as frequencies of symptom X (as present in survey data) and is purely text-based data. The data was cleaned, and the psychopathological symptoms were filtered out (biological conditions or symptoms were not included), removing all other words from each narrative. Information about the cleaning and filtration process can be found in the study that collected this data i.e., [14]. The result was a dataset with each row representing the symptoms each patient has reported, as indicated by Table 1.

The data used in the study was obtained from LiveJournal.com, where individuals have the option to share their experiences and thoughts anonymously. So, many users did not posted their private information. Additionally, not all posts included information on psychiatric diagnoses. Overall, the data on socio-demographic information or diagnostic labels is not readily available. The study by [14] highlights the unreliability of such diagnostic labels and cites extensive literature to support this. Hence, while it may be of interest to know the diagnoses of the participants, it would not be relevant to the aim of identifying correlations with auditory hallucinations and in a study where a transdiagnostic approach is being taken instead. Therefore, we have not presented the participants’ sociodemographic information, and psychiatric diagnoses here.

**Table 1:** Sample dataset (cleaned)

Patient ID	Narrative (Unfiltered)	Narrative (Filtered)
1	I feel low mood. I don’t like doing anything, just feel lethargic.	Low mood, lethargy
2	I am always having apprehensions. Often I can’t sleep at night and the lack of sleep is making it difficult for me to carry out my jobs. These days, I often feel panic attacks.	Apprehensions, lack of sleep, panic attacks
3	I used to hear voices, and that used to make me feel anxious all the time. Often I wouldn’t be able to sleep at night. But then I started my medications, and it’s much better now.	Hearing voices, anxiety, lack of sleep

For the current study, we converted this dataset by encoding the features (symptoms) using a one-hot (aka ‘one-of-K’ or ‘dummy’) encoding scheme. This results in a dataset that has rows indicating each patient. In contrast, columns indicate the symptoms they reported in binary form (where 1 represents the reporting of that symptom, while the 0 represent not reporting of that symptom). Therefore, it does not matter if the patient used the term “anxiety” ten times in their narrative. It was assigned as 1. Likewise, zero was assigned to the symptoms which a particular patient did not report in their narrative.

Together, in the resultant dataset (Table 2), each individual row in the database represents a patient (unit of analysis). Thus, thousands of columns in the database correspond to the full set of symptoms reported by all the patients in the database.

**Table 2:** Sample Data used for Pearson Correlation

Patient ID	Low Mood	Hearing Voices	Lack of sleep	…
1	1	0	0	
2	0	0	1	
3	0	1	1	

A previous study with the same dataset found that auditory hallucinations co-occur with the other symptoms in counts as enlisted in the table below in Table 3.

**Table 3:** Describing the dataset in terms of the number of narratives mentioning auditory hallucinations

Total number of symptoms reported	Total Narratives	Mentioned Auditory Hallucination	Percentage of datasets with auditory hallucination
0	704	0	0
1	964	7	0.73
2	944	6	0.64
3	791	14	1.77
4 or more	7530	597	7.93
Total	10,933	624	5.71

### Analytics procedure

The database originally had 10,933 rows representing patients and 1583 columns representing symptoms/experience words. We removed all narratives that did not have mentions of any symptoms (n = 704). One duplicate row was also removed, leaving behind 10,228 narratives in the final dataset.

Likewise, a manual scan was done to delete the useless “words” (which do not indicate symptoms or experiences). There were multiple variations of the same word, which were summed together into a single column. For example, sad and sadness were two columns, and they were clumped together to form a single column. Such preliminary cleaning process removed 136 columns, leaving 1583 words representing the symptoms or variables for the final study. In the final dataset, 624 narratives out of 10,933 narratives reported the experience of auditory hallucination (5.71%).

We performed correlation analysis to find the associates of the auditory hallucination. The current study used matplotlib.pyplot, a Python library, to implement the correlation. The aim is to find the top correlations and check if there are symptoms that were independent of auditory hallucination (i.e., correlation coefficient = 0), but in the past literature, they were suggested as correlates.

## Result

### Descriptive statistics: describing the sample of 10,228 narratives

**Fig. 1:**
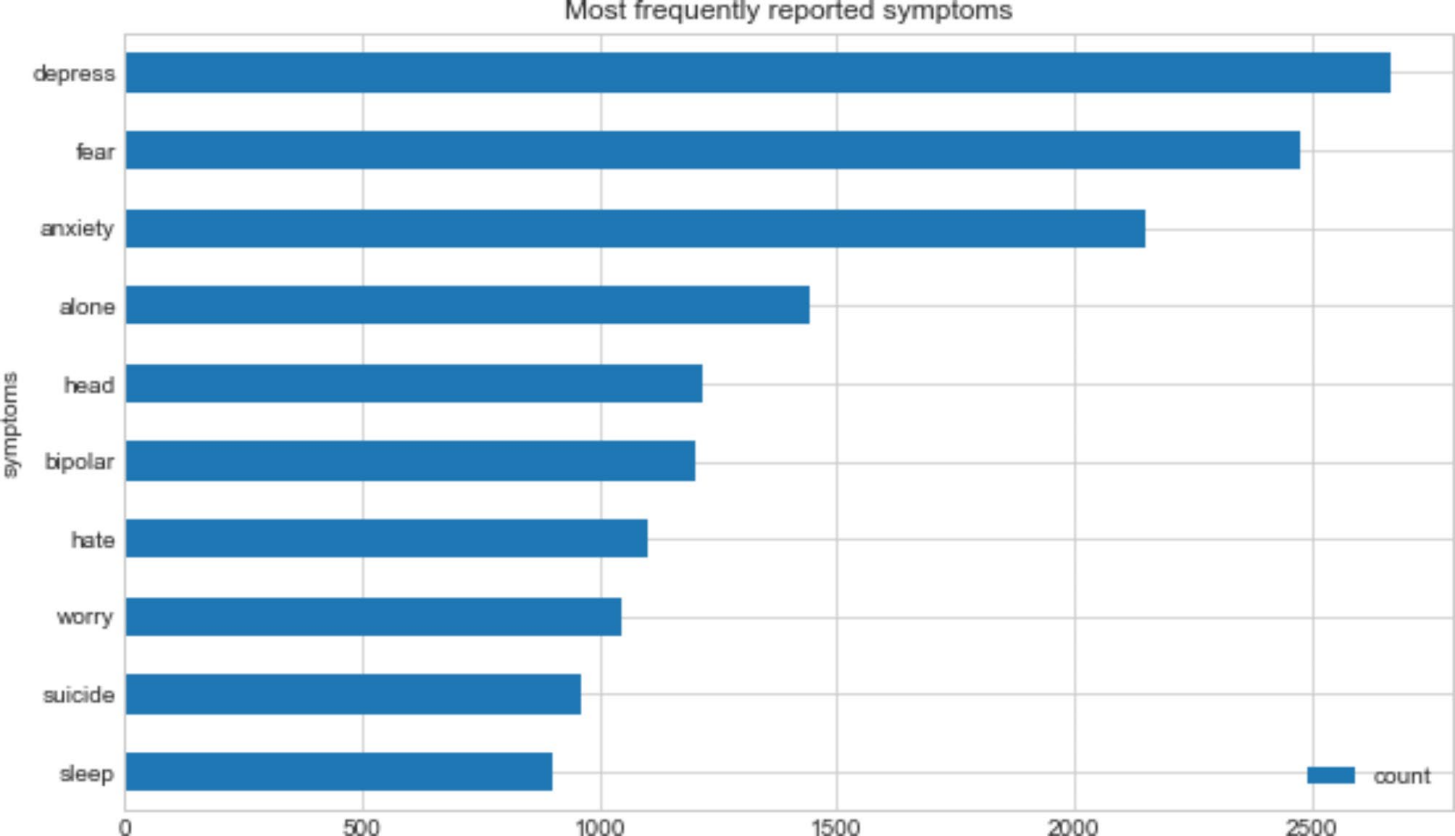
Most frequently reported symptoms (n = 10,228)

Some of the most frequently reported words in the dataset were “depression”, “anxiety”, “fear”, and “loneliness”. The Fig. 1 reports the word count for each.

### The most important correlates of auditory hallucination

The highest correlation coefficient obtained was 0.26. Using Cohen’s (1988) conventions to interpret effect size, we infer that the correlation coefficients represent a small association at best (when r = + 0.10 to + 0.30 and then from − 0.10 to -0.30). For practical reasons, we filtered out the most prominent variables to report and discuss in this study. However, the complete list is available in Supplementary Table 1 (appendix A). To filter, we chose all the correlates that had coefficients in the range of 0.10 to 0.30. Then we manually scanned them to report the most meaningful ones.

For example, the variable “head” had the third-highest correlation coefficient. Manual exploration seems to suggest, some patients reported of headaches. But since we restricted our exploration to only emotional, cognitive, behavioural experiences (and not biological/psychosomatic symptoms), so, it was not considered for further analysis.

We have reported the variables schizophrenia and psychosis, which had the 6th and 11th highest coefficients. But as mentioned above, such diagnostic terms are problematic on several grounds. For example, schizophrenia and psychosis suffer from diagnostic heterogeneity (23) both within and between individuals. This could mean that two people with very different experiences could be diagnosed with the same label.

Furthermore, such categories of mental illnesses were created and did not have ground-truth nor a biological. Hence, even when we report them, we will not discuss them further. On the other hand, individual symptoms such as delusion, paranoia, fear, suicide, and pain were reported and discussed. In total, nine variables were chosen for reporting, and 7 of them were discussed. The Table 4 below suggests correlations. Note that we did not find any correlations between − 0.10 to -0.30. All the correlates of auditory hallucination are enlisted in Supplementary Table 1 (appendix A).

**Table 4:** Pearson correlations with auditory hallucination (n = 10,228)

Correlates of Auditory Hallucinations	R	CI 95%	p-value	power
visual hallucinations	0.26	[0.24, 0.28]	< 0.0001	1
schizophrenia	0.17	[0.15, 0.19]	< 0.0001	1
fear	0.15	[0.13, 0.17]	< 0.0001	1
delusion	0.14	[0.13, 0.16]	< 0.0001	1
psychosis	0.14	[0.12, 0.15]	< 0.0001	1
paranoia	0.13	[0.11, 0.15]	< 0.0001	1
suicide	0.11	[0.09, 0.12]	< 0.0001	1
pain	0.11	[0.09, 0.12]	< 0.0001	1
trauma	0.10	[0.08, 0.12]	< 0.0001	1

From the Table 4 above, we found that visual hallucinations and auditory hallucination were positively correlated, r(10,226) = + 0.26, p < .001, CI 95% [0.24, 0.28]. Additionally, fear was also positively correlated, r(10,226) = + 0.15, p < .001, CI 95% [0.13, 0.17]. Likewise, delusion was positively correlated with auditory hallucination, r(10,226) = + 0.14, p < .001, CI 95% [0.13, 0.16]. The correlation between auditory hallucination and paranoia was positive as well, r(10,226) = + 0.13, p < .001, CI 95% [0.11, 0.15]. Moreover, the positive correlation between auditory hallucinations and suicide was r(10,226) = + 0.11, p < .001, CI 95% [0.09, 0.12]. For pain it was r(10,226) = + 0.11, p < .001, CI 95% [0.09, 0.12]. Finally, for trauma, it was r(10,226) = + 0.10, p < .001, CI 95% [0.08, 0.12].

### The variables that are independent of auditory hallucination (correlation coefficient of 0)

We scanned the 162 correlations independent of auditory hallucinations (i.e., coefficient = 0). This knowledge-based search aimed to check if any of the symptoms that the literature suggests a correlation was found to be independent of auditory hallucinations in our dataset. The complete list can be found in appendix B (Supplementary Table 2). For most of the variables, it is reasonable to expect independence from auditory hallucination. For example, hypertension (high blood pressure), nausea, weight loss and do not relate to auditory hallucinations. Therefore, in the current study, we will restrict our discussion to the variables the literature proposes to be correlated with auditory hallucinations, but we found otherwise. One such variable is dissociation.

A line of studies has proposed that dissociation plays a role in the aetiology of hallucinatory experiences (e.g., [24]). But our dataset suggests no relation. Likewise, studies suggest that the prevalence of obsessive-compulsive disorder (OCD) in individuals meeting the criteria for schizophrenia was 25% (13/52). Out of those 13 patients who met the criteria for both OCD and Schizophrenia, nine reported to experienced auditory hallucination [25]. So, accordingly, minority people who experience auditory hallucinations might also experience symptoms of OCD. Additionally, there were studies that reported a link between auditory hallucination and hearing voices e.g., [26]. Furthermore, another study reported the experience of hallucinations (including auditory-) in patients diagnosed with OCD [27]. But our findings do not suggest such a link.

## Discussion

The current study attempts to identify the strength of association between pairs of psychopathological experiences (with the constant being auditory hallucinations) while identifying their co-occurrence patterns. In the current study, we use this methodology to understand the associated experiences of auditory hallucinations better and improve our knowledge base within the psychosis and psychopathology literature.

It is not surprising to find that people who experience auditory hallucinations also report visual hallucinations. A study reviewed those 30–37% of patients with lifetime auditory hallucinations had experienced visual hallucinations, and 83–97% of patients with experience of visual hallucinations had experienced auditory hallucinations [6]. However, the mechanism is unknown. Likewise, there has been a relationship found between delusions and hallucinations. For example, [28] reported that fluctuations in the intensity (loudness and intrusiveness) of auditory hallucinations correlated closely with fluctuations in the intensity of the associated delusional beliefs, as well as with moods of anxiety and depression. One hypothesis proposed that the hallucination is the primary event, and the delusion is a secondary consequence of it [29]. In other words, the idea is that delusions arise as explanations of anomalous experiences (e.g., auditory hallucinations). The false belief is the outcome of the application of processes of inference that do not differ substantially from those generally found in non-deluded persons.

Similarly, it has been argued that auditory hallucinations precede and may trigger paranoia in people with psychosis and people at risk [30]. The exact nature of the relationship or the mechanism is yet to be determined, but a study in the past reported a relationship of auditory hallucinations and paranoia to platelet MAO activity in patients diagnosed with schizophrenia [31]. Such insights from the literature might aid in further exploration of this link. Similarly, hearing voices (auditory hallucinations) after the experience of stress (or trauma) has been reported. It has been conceptualised as a dissociative experience [32]. This might explain the link between “trauma” and “auditory hallucinations”. However, it is also possible that the experience of auditory hallucination has been reported as traumatic. Although, we argue that the first is more likely than the second. Likewise, there is evidence pertaining to the role of command hallucinations (a subtype of auditory hallucinations) in suicide [33]. In other words, patients might hear voices explicitly instructing them to engage in specified behaviours, leading to suicidal or violent acts. The connection between the two phenomena is poorly understood. However, we also found support for this relationship. Similar, we found auditory hallucinations were reported along with fear. Thus, the literature suggests that it can be a two-way relationship. For example, as mentioned above (under theoretical framework), anxiety could result from beliefs about the voices, facilitate the recurrence of auditory hallucinations (as a perpetuating factor), and anxiety can also induce verbal hallucinations [7, 10].

For some of the studies that exist, the review argued that there are methodological concerns related to the limited use of validated instruments that could pose specific barriers to the collection of meaningful phenomenological information. By using a new source of data (patient narratives) instead of survey scales and arriving at the same findings, we add additional findings to support some of the theoretical perspectives and research findings on the explanations and associates of auditory hallucinations.

Among the well-known correlations, an interesting finding from our results was the relationships between auditory hallucination and pain. There has been a report of painful somatosensory hallucinatory perceptions [34]. Still, no apparent link of pain with auditory hallucination was ever reported (to the best of our knowledge). We argue that one of the closest links with pain and auditory hallucinations was found in the association of migraines in rare cases of auditory hallucinations [35]. However, we argue that there might be an alternative mechanism(s). Further investigation might better explain the relationship between pain and auditory hallucinations.

The current study also investigated the variables independent of auditory hallucinations with a special focus on finding the ones that the literature otherwise states to have a relationship.

The current study served its purpose of finding out the correlates of auditory hallucinations. The next step would be a qualitative study that manually analyses the same dataset, reading through the narratives consisting of the correlations such as “pain” and “traumatic” – reading them will inform the exact nature of the relationship between them and auditory hallucinations. For example, narrative reading might reveal that patients who experienced auditory hallucination experienced an emotional “pain”. Thus, it might solve the current obscurity about the nature and direction of the relationship. However, such narrative analysis is beyond the scope of the current study as it requires manually reading 617 narratives that had an auditory hallucination.

A preliminary small-scale review of ten random narratives collected from individuals suffering from auditory hallucinations has revealed some intriguing aspects that underscore the significance of such datasets in understanding this phenomenon. The narratives provide a glimpse into the patients’ experiences, and several common patterns can be observed.

One of the patients complained about the inefficiencies of their local psychiatric clinic and pharmacies, highlighting the importance of addressing the practical concerns faced by individuals with mental health issues. Moreover, several individuals spoke about their belief in magic and the role it played in their lives, with some talking about their abilities of fortune telling and dreams which show them healing remedies. These instances highlight the cultural and social context in which these individuals lived, which is often neglected in the literature on mental health.

The narratives also reveal the diversity of auditory hallucinations and how they manifest differently in different individuals. For instance, one person reported hearing muffled and echoing voices from real people in front of them (even when they could hear the TV play in the adjacent room clearly), while another mentioned that they were hypersensitive to noises and found them painful. Another individual mentioned that their auditory hallucinations were restricted to their right ear. These instances suggest that auditory hallucinations may be related to changes in the brain’s auditory perception.

The narratives also shed light on the potential adaptive functions of hallucinations. Two individuals reported having visual hallucinations that were pleasant and kept them company when they felt lonely. This points to the possibility that hallucinations may serve an adaptive role and that the focus of treatment should not only be on treating the hallucinations with drugs but also on helping the person replace their imaginary friends with real-life companions.

Another pattern that emerged from the narratives was the limited conversation between the patients and their psychiatrists, which was restricted to diagnostic labels and symptoms. We argue that this potentially indicates, the biomedical approach that is prevalent in clinical practice, even when many psychiatrists claim to follow the biopsychosocial model in theory.

In conclusion, the narratives suggest that further research on the lived experiences of individuals with auditory hallucinations can provide a more nuanced understanding of this phenomenon and help redirect the focus of treatment towards a more holistic approach.

A future study dedicated to qualitatively analysing such narratives might be able to fill into the need.

In addition to highlighting such under-studied areas of the psychopathology literature, the contribution of the current study lies with the demonstration of this novel methodology. We demonstrated that it is possible to extract information from patients’ reports of their lived experiences on the internet as an economical, powerful, and a large source of existing data (so preventing the need to fund the collection of data) and further explore the data using association analysis.

### Practical implication

At times, the person might not be aware that the voices they are hearing do not have an external source. Other times, the person might not report hearing voices because they don’t want to sound “crazy” to the clinician or perceives this as a special spiritual experience (e.g., “word from God” or “being able to speak with dead people”). In all these instances, the person might not report to experience hearing voices to the clinician. But the findings from this study are expected to help clinicians identify the likelihood that the patient (reporting symptom X, Y and Z) may also be experiencing auditory hallucinations? Alternatively, when a person self-reports auditory hallucinations, knowing what other symptoms the person might be experiencing can help inform the clinical interviewing process and demonstrate a personalised, data-driven approach to an efficient triage process. This study demonstrates how text-based data can be used to derive symptom-specific information that is practical and economical. At the same time, it allows the inclusion of the voice of patients into advancing mental healthcare.

### Limitations of the current study and next steps

#### Dataset

The current study used the dataset on a heterogeneous sample. Future studies using narratives of people of a specific gender, age, and experiences might shed light on how some correlations might apply to certain demography people. It is possible that such differences in demography might have crosscut some of the effects during correlation analyses increasing the number of times such associations “might not be the case”.

The current dataset is cross-sectional. One other future possibility is to collect such narrative data on a temporal scale. The current dataset was cross-sectional and included patients who are in the early stages to people who have already recovered from their issues. Therefore, there was diversity among the narratives. Future studies could collect narrative data from patients in the same stage of their illness (e.g., people who have recovered) for homogeneity and fair comparison. Another possibility is to collect data from the same patients at different time points in their illness journey (longitudinal data). Such studies will lend insights into the trajectory of how people narrated psychopathological experiences change over time.

#### Report of obscure, generic experiences without specificity

It is to be noted that this is based on data that consisted of what patients narrated about their experiences. Therefore, it is possible that terms like depression and anxiety might not be the same as how mental health professionals think. For example, laypeople might use the word “depression” to mean low mood and likely use the word “anxiety” to mean a brief nervousness. Additionally, it is to be noted that terms like depression and anxiety do not indicate a singular experience. Instead, they are syndromes with several symptoms. Therefore, future studies are required to verify which exact symptoms of anxiety and depression were related to those who experience auditory hallucination.

#### Small coefficients

Even though we discovered and then discussed interesting links, it is to be noted that the correlation coefficients were small at best in the dataset. Given the heterogeneity of psychological experience, it is rarely the case in the real world to have all people experiencing a particular condition also report another particular condition. That is the reason why the DSM and ICD failed to reliably group people into distinct categories. But at the same time, such small correlation coefficients cannot be ignored because such symptoms are not independent and are rather dependent on each other (even if to a small extent). The current study is the first step towards exploration. Future studies might confirm the relationships we reported in this study using other methodologies such as patients’ electronic health records and survey methods.

Correlation is one approach to find associations. Future studies can utilise apriori rule mining algorithms to find similar rules and see if those can replicate the findings from this study.

## Conclusion

Overall, the correlation on narrative data described in this paper appeared to produce preliminary results about various ‘‘interesting” associations between different symptoms and Auditory Hallucinations. These promising methods have the potential to be extended and further applied. They may be able to contribute to improving our understanding of people’s subjective experiences of mental health problems which may, in turn, have important benefits for care and treatment.

## Electronic supplementary material

Below is the link to the electronic supplementary material.

Supplementary Table 1. All the variables with correlation coefficient of 0.10 to 0.30

Supplementary Table 2. List of variables which had zero correlation coefficient with auditory hallucination

## Data Availability

The data consists of sensitive information and is available on reasonable request from the corresponding author.

## References

[1] Fujita J, Takahashi Y, Nishida A, Okumura Y, Ando S, Kawano M (2015). Auditory verbal hallucinations increase the risk for suicide attempts in adolescents with suicidal ideation. Schizophr Res.

[2] Nielssen O, Bourget D, Laajasalo T, Liem M, Labelle A, Hakkanen-Nyholm H (2009). Homicide of Strangers by people with a psychotic illness. Schizophr Bull.

[3] Shinn A, Pfaff D, Young S, Lewandowski K, Cohen B, Öngür D (2012). Auditory hallucinations in a cross-diagnostic sample of psychotic disorder patients: a descriptive, cross-sectional study. Compr Psychiatr.

[4] Boele F, Rooney A, Grant R, Klein M. Psychiatric symptoms in glioma patients: from diagnosis to management. Neuropsychiatr Dis Treat. 2015;1413. 10.2147/ndt.s65874. 10.2147/NDT.S65874 PMC4467748 26089669

[5] Dhossche D, Ferdinand R, Van Der Ende J, Hofstra M, Verhulst F (2002). Diagnostic outcome of self-reported hallucinations in a community sample of adolescents. Psychol Med.

[6] McCarthy-Jones S, Smailes D, Corvin A, Gill M, Morris D, Dinan T (2017). Occurrence and co-occurrence of hallucinations by modality in schizophrenia-spectrum disorders. Psychiatry Res.

[7] van der Gaag M, Hageman M, Birchwood M (2003). Evidence for a cognitive model of auditory hallucinations. J Nerv Ment Dis.

[8] Chadwick P, Birchwood M (1994). The omnipotence of Voices. Br J Psychiatry.

[9] Waters F, Allen P, Aleman A, Fernyhough C, Woodward T, Badcock J (2012). Auditory hallucinations in Schizophrenia and Nonschizophrenia populations: a review and Integrated Model of Cognitive Mechanisms. Schizophr Bull.

[10] Ratcliffe M, Wilkinson S (2016). How anxiety induces verbal hallucinations. Conscious Cogn.

[11] Yung A, McGorry P (1996). The Prodromal phase of first-episode psychosis: past and current conceptualizations. Schizophr Bull.

[12] Yung A, Phillips L, Yuen H, Francey S, McFarlane C, Hallgren M, McGorry P (2003). Psychosis prediction: 12-month follow up of a high-risk (“prodromal”) group. Schizophr Res.

[13] Fava G, Guidi J, Grandi S, Hasler G (2014). The Missing Link between Clinical States and biomarkers in Mental Disorders. Psychother Psychosom.

[14] Ghosh C, McVicar D, Davidson G, Shannon C. Measuring diagnostic heterogeneity using text-mining of the lived experiences of patients. BMC Psychiatry. 2021;21(1). 10.1186/s12888-021-03044-1. 10.1186/s12888-021-03044-1 PMC7842026 33509154

[15] Ghosh C, McVicar D, Davidson G, Shannon C, Armour C. What can we learn about the psychiatric diagnostic categories by analysing patients’ lived experiences with Machine-Learning? BMC Psychiatry. 2022;22(1). 10.1186/s12888-022-03984-2. 10.1186/s12888-022-03984-2 PMC9233399 35751077

[16] Fried E, Nesse R. Depression sum-scores don’t add up: why analyzing specific depression symptoms is essential. BMC Med. 2015;13(1). 10.1186/s12916-015-0325-4. 10.1186/s12916-015-0325-4 PMC4386095 25879936

[17] Armour C, Fried E, Olff M (2017). PTSD symptomics: network analyses in the field of psychotraumatology. Eur J Psychotraumatology.

[18] Armour C, Fried E, Deserno M, Tsai J, Pietrzak R (2017). A network analysis of DSM-5 posttraumatic stress disorder symptoms and correlates in U.S. military veterans. J Anxiety Disord.

[19] Beard C, Millner A, Forgeard M, Fried E, Hsu K, Treadway M (2016). Network analysis of depression and anxiety symptom relationships in a psychiatric sample. Psychol Med.

[20] Cramer A, Waldorp L, van der Maas H, Borsboom D (2010). Co-morbidity: a network perspective. Behav Brain Sci.

[21] Fried E (2017). The 52 symptoms of major depression: lack of content overlap among seven common depression scales. J Affect Disord.

[22] Kreitchmann R, Abad F, Ponsoda V, Nieto M, Morillo D. Controlling for Response Biases in Self-Report Scales: forced-choice vs. psychometric modeling of likert items. Front Psychol. 2019;10. 10.3389/fpsyg.2019.02309. 10.3389/fpsyg.2019.02309 PMC6803422 31681103

[23] Tsuang M, Lyons M, Faraone S (1990). Heterogeneity of Schizophrenia. Br J Psychiatry.

[24] Varese F, Udachina A, Myin-Germeys I, Oorschot M, Bentall R (2011). The relationship between dissociation and auditory verbal hallucinations in the flow of daily life of patients with psychosis. Psychosis.

[25] Tibbo P, Kroetsch M, Chue P, Warneke L (2000). Obsessive–compulsive disorder in schizophrenia. J Psychiatr Res.

[26] Morrison AP, Baker CA (2000). Intrusive thoughts and auditory hallucinations: a comparative study of intrusions in psychosis. Behav Res Ther.

[27] Fontenelle LF, Hasler G (2008). The analytical epidemiology of obsessive-compulsive disorder: risk factors and correlates. Progress in neuro-psychopharmacology & biological psychiatry.

[28] Hustig H, Hafner R (1990). Persistent auditory hallucinations and their relationship to delusions and Mood. J Nerv Ment Dis.

[29] Maher B (2006). The relationship between delusions and hallucinations. Curr Psychiatry Rep.

[30] Schlier B, Lincoln T. (2017). Do hallucinations predict paranoia or vice versa? Results from two ambulatory assessment studies. In *Conference: 6th European Conference on Schizophrenia Research (ECSR)*.

[31] Meltzer H, Zureick J (1987). Relationship of auditory hallucinations and paranoia to platelet MAO activity in schizophrenics: sex and race interactions. Psychiatry Res.

[32] Clifford G, Dalgleish T, Hitchcock C (2018). Prevalence of auditory pseudohallucinations in adult survivors of physical and sexual trauma with chronic post-traumatic stress disorder (PTSD). Behav Res Ther.

[33] Montross LP, Zisook S, Kasckow J. Command hallucinations and suicide risk. In: Tatarelli R, Pompili M, Girardi P, editors. Suicide in schizophrenia. Nova Biomedical Books; 2007. pp. 113–32.

[34] Bär K, Gaser C, Nenadic I, Sauer H (2002). Transient activation of a somatosensory area in painful hallucinations shown by fMRI. NeuroReport.

[35] Miller E, Grosberg B, Crystal S, Robbins M (2014). Auditory hallucinations associated with migraine: Case series and literature review. Cephalalgia.

