# Supplementary material for: Exploring the associations between auditory hallucinations and psychopathological experiences in 10,933 patient narratives: moving beyond diagnostic categories and surveys
Source: BMC Psychiatry. 2023 May 2;23:307. doi: 10.1186/s12888-023-04780-2 (PMC10155450; doi:10.1186/s12888-023-04780-2)
Supplement: Supplementary file 1 — Supplementary Table 1. All the variables with correlation coefficient of 0.10 to 0.30 [file 12888_2023_4780_MOESM1_ESM.docx]

**Appendix A**

Supplementary Table 1. All the variables with correlation coefficient of 0.10 to 0.30.

| Correlates | auditory-hallucinations |
| --- | --- |
| auditory-hallucinations | 1.00 |
| visual-hallucinations | 0.26 |
| head | 0.19 |
| deductible | 0.19 |
| hallucinations | 0.18 |
| schizophrenia | 0.17 |
| fear | 0.15 |
| delusion | 0.14 |
| hearing | 0.14 |
| speak | 0.14 |
| psychosis | 0.14 |
| paranoia | 0.13 |
| alone | 0.13 |
| reality | 0.12 |
| following | 0.11 |
| dangerous | 0.11 |
| visions | 0.11 |
| struggle | 0.11 |
| screaming | 0.11 |
| brain | 0.11 |
| anger | 0.11 |
| suicide | 0.11 |
| pain | 0.11 |
| inner | 0.10 |
| face | 0.10 |
| psychotic | 0.10 |
| control | 0.10 |
| trauma | 0.10 |
| recognize | 0.10 |
