# Supplementary material for: Exploring the associations between auditory hallucinations and psychopathological experiences in 10,933 patient narratives: moving beyond diagnostic categories and surveys
Source: BMC Psychiatry. 2023 May 2;23:307. doi: 10.1186/s12888-023-04780-2 (PMC10155450; doi:10.1186/s12888-023-04780-2)
Supplement: Supplementary file 2 — Supplementary Table 2. List of variables which had zero correlation coefficient with auditory hallucination [file 12888_2023_4780_MOESM2_ESM.docx]

**Appendix B**

Supplementary Table 2. List of variables which had zero correlation coefficient with auditory hallucination

| Variables or words independent of auditory hallucination | Correlation Coefficient |
| --- | --- |
| burn | 0.00 |
| size | 0.00 |
| tied | 0.00 |
| disaster | 0.00 |
| rationally | 0.00 |
| relaxation | 0.00 |
| sensations | 0.00 |
| shots | 0.00 |
| irritating | 0.00 |
| veterans | 0.00 |
| self-help | 0.00 |
| bitter | 0.00 |
| dull | 0.00 |
| flu | 0.00 |
| porn | 0.00 |
| guarded | 0.00 |
| fright | 0.00 |
| accidents | 0.00 |
| obsessions-compulsions | 0.00 |
| instability | 0.00 |
| dragging | 0.00 |
| separation | 0.00 |
| dissociation | 0.00 |
| purging | 0.00 |
| thin | 0.00 |
| heat | 0.00 |
| marry | 0.00 |
| uneasy | 0.00 |
| roommates | 0.00 |
| relapsed | 0.00 |
| social-anxiety | 0.00 |
| antidepressant | 0.00 |
| nausea | 0.00 |
| generalized | 0.00 |
| dragon | 0.00 |
| tiredness | 0.00 |
| sleepless | 0.00 |
| addictions | 0.00 |
| charged | 0.00 |
| interacting | 0.00 |
| harming | 0.00 |
| messed | 0.00 |
| weight-loss | 0.00 |
| hideous | 0.00 |
| spine | 0.00 |
| unconscious | 0.00 |
| insensitive | 0.00 |
| bothering | 0.00 |
| sickness | 0.00 |
| panicked | 0.00 |
| prozac | 0.00 |
| anti-anxiety | 0.00 |
| socialize | 0.00 |
| anti-psychotics | 0.00 |
| ssri | 0.00 |
| germs | 0.00 |
| obsessions | 0.00 |
| triggering | 0.00 |
| tablet | 0.00 |
| socialanxiety | 0.00 |
| spider | 0.00 |
| sedative | 0.00 |
| methadone | 0.00 |
| depressing | 0.00 |
| spouse | 0.00 |
| tempting | 0.00 |
| catatonia | 0.00 |
| cogentin | 0.00 |
| ankle | 0.00 |
| topomax | 0.00 |
| addict | 0.00 |
| adderal | 0.00 |
| weed | 0.00 |
| cocaine | 0.00 |
| sweat | 0.00 |
| headache | 0.00 |
| offended | 0.00 |
| tolerance | 0.00 |
| panicky | 0.00 |
| cuts | 0.00 |
| freeze | 0.00 |
| cramps | 0.00 |
| obsessive-compulsive | 0.00 |
| lexapro | 0.00 |
| troubles | 0.00 |
| generalised-anxiety | 0.00 |
| over-weight | 0.00 |
| slipped | 0.00 |
| purge | 0.00 |
| polar | 0.00 |
| wars | 0.00 |
| awkwardness | 0.00 |
| helplessness | 0.00 |
| kidney | 0.00 |
| apprehensive | 0.00 |
| o_c_d | 0.00 |
| stroke | 0.00 |
| addicted | 0.00 |
| fiance | 0.00 |
| overwhelmingly | 0.00 |
| relaxing | 0.00 |
| tearful | 0.00 |
| knee | 0.00 |
| ecstatic | 0.00 |
| distract | 0.00 |
| infection | 0.00 |
| bleed | 0.00 |
| prejudices | 0.00 |
| ors | 0.00 |
| antisocial-personality | 0.00 |
| cynical | 0.00 |
| bomb | 0.00 |
| chew | 0.00 |
| ssi | 0.00 |
| valium | 0.00 |
| vacation | 0.00 |
| fired | 0.00 |
| stabilizer | 0.00 |
| kindergarten | 0.00 |
| rash | 0.00 |
| flashback | 0.00 |
| dope | 0.00 |
| cheated | 0.00 |
| beast | 0.00 |
| gross | 0.00 |
| anniversary | 0.00 |
| starving | 0.00 |
| bruise | 0.00 |
| grumpy | 0.00 |
| expansive | 0.00 |
| nonverbal | 0.00 |
| pessimism | 0.00 |
| hypochondriasis | 0.00 |
| woe | 0.00 |
| reconciliation | 0.00 |
| constipation | 0.00 |
| methamphetamine | 0.00 |
| grouchy | 0.00 |
| constipated | 0.00 |
| fugue | 0.00 |
| recklessness | 0.00 |
| luvox | 0.00 |
| binge | 0.00 |
| cbt | 0.00 |
| surgery | 0.00 |
| wan | 0.00 |
| self-conscious | 0.00 |
| lethargic | 0.00 |
| reject | 0.00 |
| ful | 0.00 |
| underactive | 0.00 |
| mutism | 0.00 |
| worthless | 0.00 |
| fidget | 0.00 |
| somatic | 0.00 |
| dysthymic | 0.00 |
| bowels | 0.00 |
| grossly | 0.00 |
| premenstrual | 0.00 |
| hating | 0.00 |
| nervousness | 0.00 |
